# Rapid 4D-MRI reconstruction using a deep radial convolutional neural network: Dracula

**DOI:** 10.1016/j.radonc.2021.03.034

**Published:** 2021-06

**Authors:** Joshua N. Freedman, Oliver J. Gurney-Champion, Simeon Nill, Anna-Maria Shiarli, Hannah E. Bainbridge, Henry C. Mandeville, Dow-Mu Koh, Fiona McDonald, Marc Kachelrieß, Uwe Oelfke, Andreas Wetscherek

**Affiliations:** a Joint Department of Physics, The Institute of Cancer Research and The Royal Marsden NHS Foundation Trust, London, United Kingdom; b Department of Radiology and Nuclear Medicine, Cancer Center Amsterdam, Amsterdam UMC, University of Amsterdam, The Netherlands; c Department of Radiotherapy, The Institute of Cancer Research and The Royal Marsden NHS Foundation Trust, London, United Kingdom; d Department of Radiotherapy, Portsmouth Hospitals University NHS Trust, Queen Alexandra Hospital, United Kingdom; e Department of Radiology, The Royal Marsden NHS Foundation Trust, London, United Kingdom; f Division of X-Ray Imaging and CT, German Cancer Research Center (DKFZ), Heidelberg, Germany

**Keywords:** 4D MRI, Deep convolutional neural networks, Radiotherapy treatment planning, Magnetic resonance guided radiotherapy, MR-Linac

## Abstract

**Background and Purpose:**

4D and midposition MRI could inform plan adaptation in lung and abdominal MR-guided radiotherapy. We present deep learning-based solutions to overcome long 4D-MRI reconstruction times while maintaining high image quality and short scan times.

**Methods:**

Two 3D U-net deep convolutional neural networks were trained to accelerate the 4D joint MoCo-HDTV reconstruction. For the first network, gridded and joint MoCo-HDTV-reconstructed 4D-MRI were used as input and target data, respectively, whereas the second network was trained to directly calculate the midposition image. For both networks, input and target data had dimensions of 256 × 256 voxels (2D) and 16 respiratory phases. Deep learning-based MRI were verified against joint MoCo-HDTV-reconstructed MRI using the structural similarity index (SSIM) and the naturalness image quality evaluator (NIQE). Moreover, two experienced observers contoured the gross tumour volume and scored the images in a blinded study.

**Results:**

For 12 subjects, previously unseen by the networks, high-quality 4D and midposition MRI (1.25 × 1.25 × 3.3 mm^3^) were each reconstructed from gridded images in only 28 seconds per subject. Excellent agreement was found between deep-learning-based and joint MoCo-HDTV-reconstructed MRI (average SSIM ≥ 0.96, NIQE scores 7.94 and 5.66). Deep-learning-based 4D-MRI were clinically acceptable for target and organ-at-risk delineation. Tumour positions agreed within 0.7 mm on midposition images.

**Conclusion:**

Our results suggest that the joint MoCo-HDTV and midposition algorithms can each be approximated by a deep convolutional neural network. This rapid reconstruction of 4D and midposition MRI facilitates online treatment adaptation in thoracic or abdominal MR-guided radiotherapy.

## Highlights

• Deep learning accelerates 4D-MRI recon for online adaptive MR-guided radiotherapy.
• First reconstruction of high resolution whole thorax 4D-MRI for 16 phases in 28 s.
• First use of dCNNs for midposition reconstruction from undersampled 4D-MRI in 28 s.
• Excellent agreement between deep learning-based tumour midposition and reference.

Magnetic resonance (MR) guided radiotherapy (MRgRT) harnesses the exquisite soft-tissue contrast of magnetic resonance imaging (MRI) to improve the conformity of radiotherapy treatment delivery and subsequently patient outcome [1], [2], [3]. For abdominal and lung cancer patients, four-dimensional (4D) and midposition [4], [5] (MidP; time-weighted mean position within respiratory cycle) MRI have many applications on an MR-Linac [6], [7], [8], [9], [10], [11], [12], [13], [14]. For instance, 4D/MidP-MRI could be acquired *online* before treatment delivery (pre-beam) and inform treatment plan adaptation to better represent the daily patient anatomy and respiratory pattern [15], [16], [17]. Alternatively, MidP-MRI could be calculated from pre-beam 4D-MRI and then continuously updated throughout treatment delivery based on 2D cine MRI, to adapt, for example, to baseline drifts [18], [19].

To achieve reasonable scanning times, 4D-MRI data are acquired heavily undersampled and iterative compressed sensing-based reconstructions [20], [21], [22] are used to calculate high-quality 4D/MidP-MRI. However, state-of-the-art reconstructions are currently of limited use for online MRgRT applications because of long reconstruction times. For the 4D joint motion-compensated high-dimensional total variation (joint MoCo-HDTV) algorithm, 9–12 h were reported [23], while current implementations of the Golden-angle Radial Sparse Parallel (GRASP) reconstruction take approximately 10 min [20], [24], [25].

Deep learning techniques can bypass the long calculation times required by iterative compressed sensing algorithms to reconstruct highly undersampled data [26], [27], [28], [29], [30]. Deep learning-based MR image reconstruction techniques can be loosely categorised into data-driven, model-based and hybrid methods [31].

In data-driven approaches, a mapping is obtained between input and target data using a deep convolutional neural network (dCNN) architecture, with either minor or no use of a priori information. For instance, Han et al. applied a dCNN to reconstruct undersampled two-dimensional (2D) MR images [32], which were acquired using a radial stack-of-stars spoiled gradient echo sequence [33]. Similarly, Hyun et al. trained and applied a dCNN to reduce aliasing artefacts in 2D-MR images [34], which were acquired using a Cartesian turbo spin echo sequence.

In model-based techniques, image reconstruction is formulated as an iterative optimisation problem with free parameters and functions. By unrolling the optimisation algorithm, on an iteration by iteration basis, a deep network can be constructed and trained to perform image reconstruction [28]. Hybrid approaches combine data-driven and model-based techniques. For example, [29], [35] proposed frameworks utilising dCNN-based regularizers (data-driven) and data consistency steps (model-based) to reconstruct undersampled MR images.

From the literature, it is clear that dCNNs can rapidly reduce aliasing artefacts present in undersampled MR images [30], [31], [32]. However, dCNNs have not yet been applied to accelerate compressed sensing-based 4D/MidP-MRI reconstructions.

In this article, data-driven dCNNs were implemented, trained and applied to reconstruct 4D-MRI and MidP-MRI from gridded data. The implemented architecture is referred to as Dracula, i.e. a **d**eep **ra**dial **c**onvol**u**tiona**l** neur**a**l network. The dCNN-reconstructed images were verified against 4D joint MoCo-HDTV-reconstructed MRI by comparison of image similarity (structural similarity index metric (SSIM) and naturalness image quality evaluator (NIQE) [36]). In addition, dCNN-reconstructed 4D-MRI were visually assessed and compared against joint MoCo-HDTV-reconstructed 4D-MRI by two experienced observers.

## Materials and methods

### Data acquisition

Within imaging studies approved by the local ethics committee (16/LO/0591 and 16/LO/1390), 20 adult healthy volunteers and 47 patients (27 lung cancer [adult]; 3 liver cancer [adult]; 17 abdominal cancer [paediatric]) were scanned in free breathing with a volumetric T1-weighted radial stack-of-stars spoiled gradient echo sequence [33] with golden-angle spacing [37] at 1.5 T (MAGNETOM Aera; Siemens Healthcare; Erlangen, DE). T1-weighted images were acquired in axial orientation with pixel-size 1.25 × 1.25–1.50 × 1.50 mm^2^ and slice-thickness 3.0–3.5 mm. Written informed consent was obtained for all patients to use their images for research purposes. Very young patients were scanned under general anaesthesia and required a smaller imaging field-of-view. Detailed acquisition parameters are provided in the supplemental material.

### Reconstruction of raw data

Raw data were corrected for gradient-delays [38] and sorted into 16 overlapping respiratory phases using a self-gating signal [39]. Afterwards, the joint MoCo-HDTV algorithm was applied to calculate high-quality 4D magnitude images from the sorted raw data [23], which will onwards be referred to as 4D-MoCo. In addition, 4D-Gridded images were obtained from the sorted raw data by density compensation and interpolation onto a rectilinear grid using a Kaiser–Bessel kernel followed by a fast Fourier transform [23]. Removal of oversampling resulted in a matrix size of 256 × 256 pixels for each slice for both 4D-Gridded and 4D-MoCo MRI.

### Calculation of midposition images

Midposition images were obtained from 4D-MoCo MRI using a similar approach to [40]. Motion vector fields (MVFs) $\left(M_{T_{n}}^{T_{1}}\right)$ between the end-exhalation phase $\left(T_{1}\right)$ and all other respiratory phases ($T_{n})$ of 4D-MoCo MRI were calculated by b-spline GPU-accelerated deformable image registration using NiftyReg [41], [42]. The average transformation $\left(M_{T_{\mathrm{MidP}}}^{T_{1}}\right)$ was then calculated from the $M_{T_{n}}^{T_{1}}$ set [4]. Next, MVFs describing the transformation between MidP and all respiratory phases ($T_{n}$) were obtained by composing the $M_{T_{n}}^{T_{1}}$ and inverse $M_{T_{\mathrm{MidP}}}^{T_{1}}$MVFs: $M_{T_{n}}^{T_{\mathrm{MidP}}}=M_{T_{n}}^{T_{1}}{}^{\circ}M_{T_{1}}^{T_{\mathrm{MidP}}}$. Afterwards, the MidP-MoCo image was calculated by warping all $T_{n}$ with the inverse $M_{T_{n}}^{T_{\mathrm{MidP}}}$ transformations and averaging over all respiratory phases: MidP-MoCo$=\frac{1}{16}\sum_{k=1}^{16}M_{T_{\mathrm{MidP}}}^{T_{k}}T_{k}$.

### Data pre-processing

Per subject, the intensity values of the MidP-MoCo, 4D-Gridded and 4D-MoCo images were divided by 1.5 times the maximum intensity of the 4D-Gridded image. Scaling ensured that image intensity values were between 0 and 1.

Scaled data were randomly assigned to training (50 subjects [75%]), validation (11 subjects [16%]) and test sets (6 subjects [9%]); collectively referred to as Group 1. Random sorting was constrained to ensure that ratios between the number of healthy volunteers, lung cancer patients, liver cancer patients and abdominal cancer patients were similar in the training, validation and test sets. Using the same approach, a second data set (Group 2) was obtained. Test sets of Group 1 and 2 were disjoint, each containing data from two healthy volunteers, three lung cancer patients and one abdominal cancer patient.

### Implementation of Dracula

**Fig. 1:**
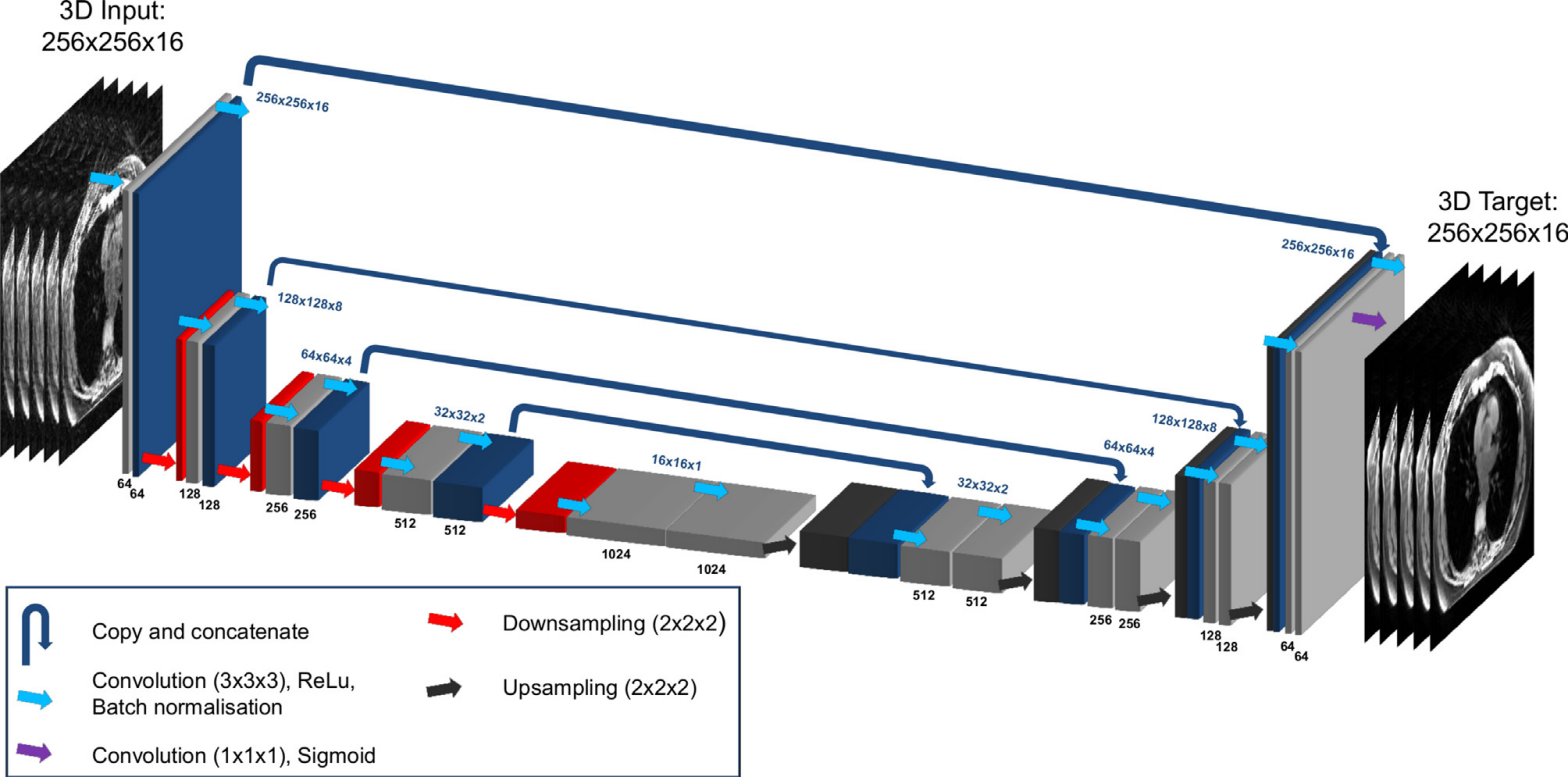
Implemented Dracula network architecture. Black and blue numbers display the number and matrix-size of feature maps at each stage of the network. Dracula was trained using Gridded (Input) and MoCo (Target) reconstructed images of dimensions: 256 [voxels] × 256 [voxels] × 16 [respiratory phases].

A three-dimensional (3D) U-net dCNN architecture [43], [44] was implemented in TensorFlow [45] to separately reconstruct 4D-MoCo and MidP-MoCo images from 4D-Gridded MRI. The output image volumes are referred to as 4D-Dracula and MidP-Dracula, respectively. Fig. 1 displays the Dracula architecture, which contained encoding (left-hand side), bottleneck (middle) and decoding (right-hand side) paths. Overall, Dracula contained 90,304,449 trainable parameters, which were associated with batch normalisation (shifting and scaling parameters), convolutions (kernel weights) and transposed convolutions (kernel weights). Further information on the choices regarding the implemented network architecture and how overfitting is prevented, is provided as supplemental material.

### Training and application of Dracula

Using the training and validation data from Group 1, two separate instances of Dracula were optimised to learn transformations between the following input and target images:

**Dracula-4D-1:** Generates 4D-Dracula images from 4D-Gridded data.
**Dracula-MidP-1:** Generates MidP-Dracula images from 4D-Gridded data.

For both networks, input and target images had dimensions 256 [voxels] × 256 [voxels] × 16 [respiratory phases] to enable capturing of features at different length scales and to resolve motion between respiratory phases. For the Dracula-MidP-1 network, copies of the MidP-MoCo image were used as the target image for each of the 16 respiratory phases.

**Fig. 2:**
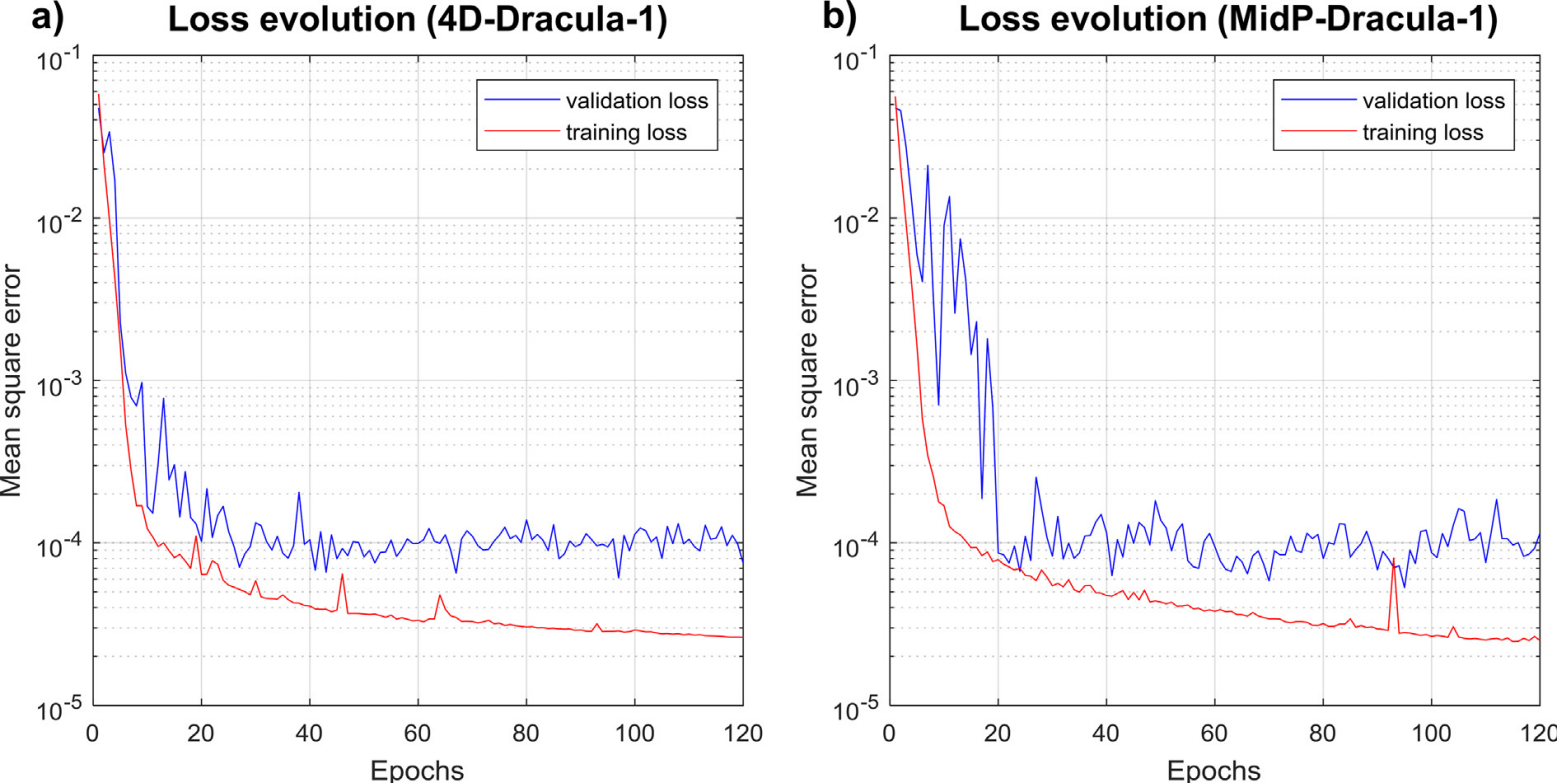
Training curves demonstrating the optimizer loss convergence for the a) Dracula-4D-1 and b) Dracula-MidP-1 networks. In both cases, the validation loss was stable and had converged between 20 and 30 epochs.

All networks were trained using the Adam optimizer [46] with mean square error as the loss function and hyper-parameters: learning rate = 10^−5^, epochs = 120 and batch-size = 1 (GPU memory constraints). Hyper-parameters were empirically optimised by monitoring the training and validation loss curves (Fig. 2a) and by manually assessing the image quality of 4D-Dracula images generated from the validation data at subsequent epochs throughout training. While the validation loss curves were stable after epochs 20–30, slight improvements in image quality were observed in the validation images up to epoch 120. Identical hyper-parameters were chosen for the Dracula-MidP-1 network as the resulting training and validation loss curves exhibited similar behaviour to those corresponding to the Dracula-4D-1 network (Fig. 2b).

For cross-validation purposes, two further instances of Dracula (Dracula-4D-2 and Dracula-MidP-2) were trained in the same way as the Dracula-4D-1 and Dracula-MidP-1 networks, except using training and validation data from Group 2.

Once trained, all networks were separately applied to reconstruct test data from Groups 1 (Dracula-4D-1 and Dracula-MidP-1) and 2 (Dracula-4D-2 and Dracula-MidP-2). Test data were previously unseen by the networks. All training and testing calculations were performed on an NVIDIA Quadro P6000 GPU with 24 GB memory.

MidP-Dracula MRI was obtained from the output of the Dracula-MidP networks by averaging over the respiratory phase dimension.

### Verification of Dracula-reconstructed images

4D-Dracula and MidP-Dracula MRI were verified against the corresponding 4D-MoCo and MidP-MoCo images. Heavy streaking artefacts exhibited by 4D-Gridded MRI prohibited calculation of and comparison to a MidP-Gridded image. Images were compared in terms of the SSIM [47] and NIQE [36] metrics. The SSIM is scaled between 0 and 1, where the SSIM of two images = 1, if they are identical in terms of contrast, luminance and structure. The NIQE score measures the distance between the natural scene statistic (NSS) features of an image and a pre-trained model. Lower NIQE scores symbolise better agreement with the model in terms of perceptual image quality. Here, we trained an NIQE model using 1000 randomly extracted 2D slices from the 4D-MoCo test data of Groups 1 and 2.

A radiation oncologist and a radiologist independently evaluated the exhalation and inhalation respiratory phase images of 4D-Gridded, 4D-MoCo and 4D-Dracula MRI (Groups 1 and 2: 8 patients and 4 healthy volunteers). Both observers had at least five years of experience reading abdominal and thoracic MR images. All images were anonymized and randomly presented in RayStation software (v7.99, RaySearch Laboratories, Sweden). Images were scored using a five-point Likert scale: 0 – unreadable, 1 – poor, 2 – adequate (clinically acceptable for target and organ-at-risk delineation in radiotherapy treatment planning), 3 – good and 4 – excellent. Images were assessed in terms of: general image sharpness, general streaking artefacts, visibility of the tumour extent, visibility of the heart and visibility of the oesophagus. It was not possible to assess the visibility of the heart and the oesophagus for two abdominal cancer patients because these organs were outside the field-of-view. Moreover, the visibility of the tumour extent was not scored for three patients (two abdominal and one lung) because the gross tumour volume (GTV) could not be localised without additional clinical information. Independently for observers 1 and 2, scores given to each reconstruction algorithm were compared using a two-sided paired Wilcoxon signed-rank test (*α* = 0.05). We report mean and standard deviation over all test-subjects for each reconstruction and scoring category. In addition, the inter-observer agreement was assessed for each metric using the intra class correlation coefficient (ICC) in R [48] with *α* = 0.05, two-way mixed effects, single measures and consistency agreement. Based on [49], scores 0.00–0.39, 0.40–0.59, 0.60–0.74 and 0.75–1.00 were interpreted as poor, fair, good and excellent, respectively.

Both observers delineated the GTV for five patients to enable a comparison of the tumour motion range and contour similarity between the 4D-Dracula and 4D-MoCo images. Tumour motion range was calculated as the Euclidean distance between the GTV centre-of-mass in exhalation and inhalation. Motion ranges for 4D-Dracula and 4D-MoCo were compared using a two-sided paired Wilcoxon signed-rank test (*α* = 0.05). Separately for each patient and observer, the Sørensen-Dice coefficient (DSC) [50] was calculated between GTV contours delineated on corresponding phases of 4D-Dracula and 4D-MoCo images. Further, to evaluate inter-observer contour variation, the DSC coefficients of GTV contours delineated on the same images were calculated.

Tumour positions in MidP-Dracula were verified against MidP-MoCo using image registration. For each patient, from the GTV contour on the 4D-MoCo exhalation image, a rectangular bounding box was obtained after morphological dilatation with a spherical structuring element (3, 3, 3 pixels) and used as a mask to extract the tumour region. The difference of the tumour position between MidP-Dracula and the masked MidP-MoCo image was then obtained by rigid registration using the imregister function in MATLAB (version 2020b; The Mathworks, Natik, MA).

## Results

**Fig. 3:**
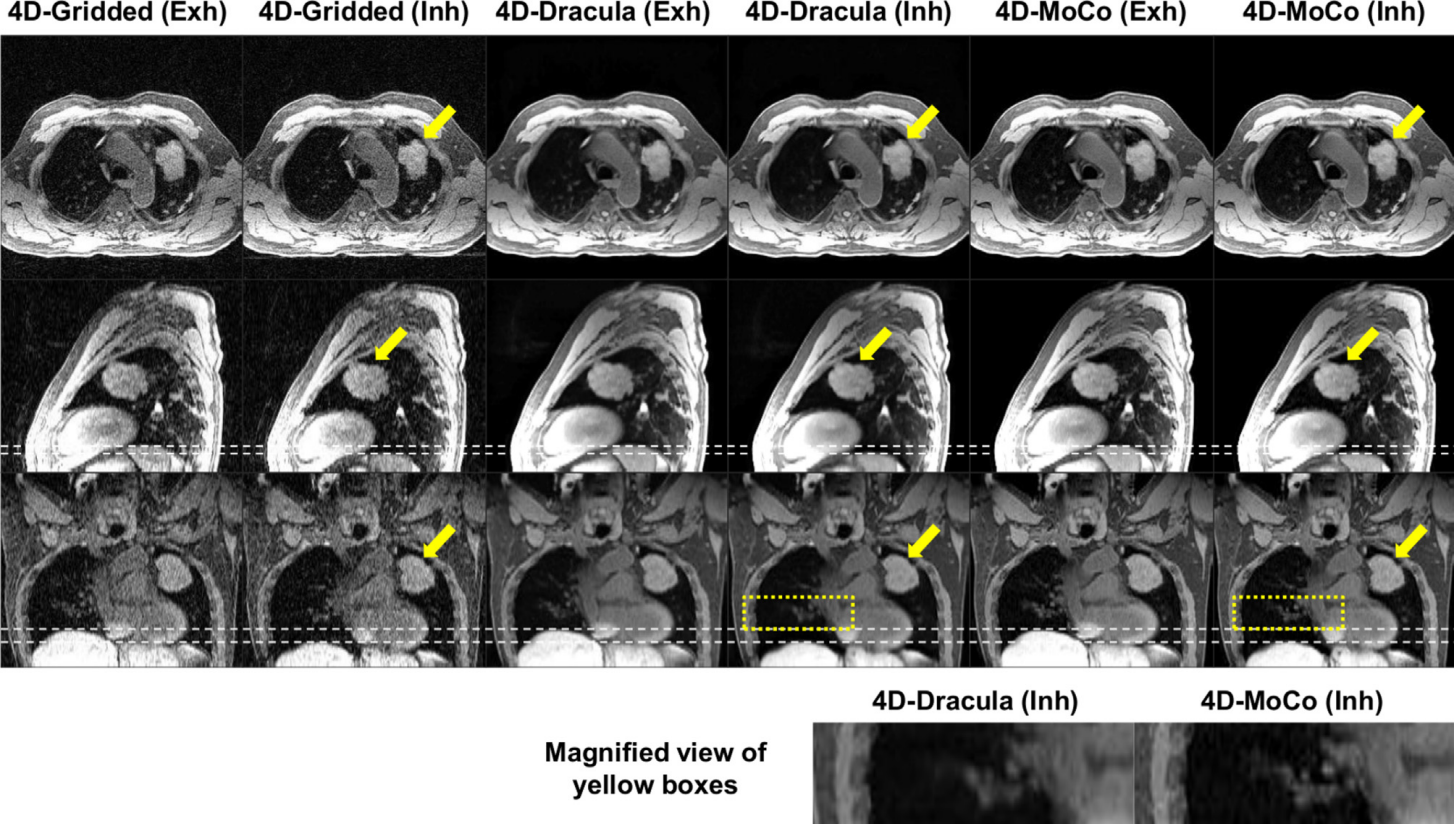
Comparison of the exhalation (Exh) and inhalation (Inh) respiratory phases of the 4D-Gridded, 4D-Dracula and 4D-MoCo reconstructed images of a representative lung cancer patient. Dracula restores image quality in a comparable manner to the joint MoCo-HDTV reconstruction with only minor blurring; e.g. see the magnified view of the region surrounded by the yellow boxes. For this patient, the average 4D-Gridded, 4D-Dracula and 4D-MoCo observer scores were 1.3, 3.3 and 4.0 (general image sharpness), 1.0, 3.5 and 4.0 (general streaking artefacts), 1.0, 3.75 and 4.0 (visibility of the tumour extent), 1.75, 3.5 and 4.0 (visibility of the heart) and 0.75, 3.0, 3.5 (visibility of the oesophagus), respectively. White dashed lines aid comparison of the diaphragm position. Yellow arrows point to the tumour site.

**Fig. 4:**
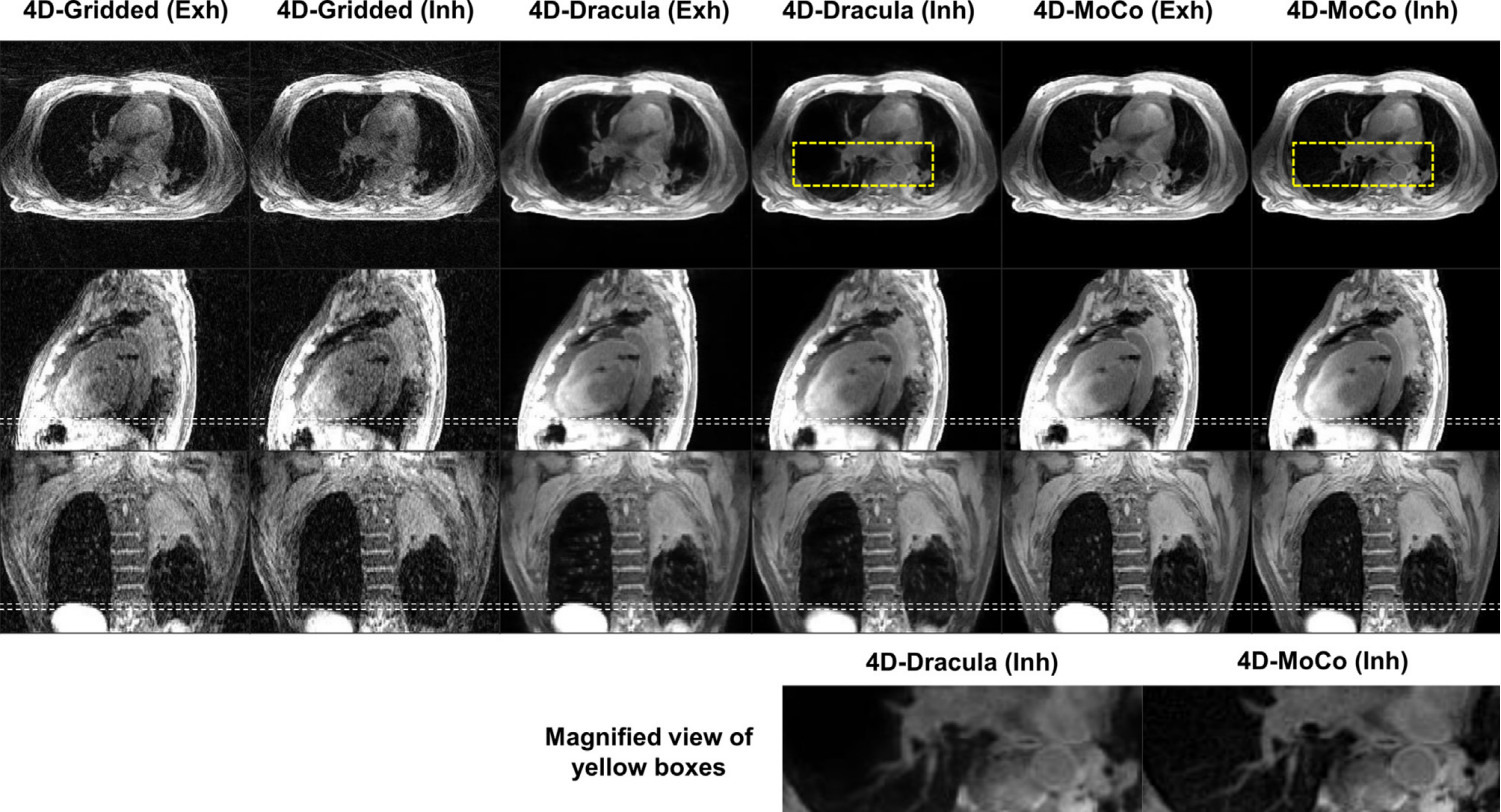
Comparison of exhalation (Exh) and inhalation (Inh) phases for the lung cancer patient with the lowest overall observer scores. Dracula achieves clinically acceptable image quality, but minor to moderate blurring remains. See, for instance, the vessels inside the lung and the oesophagus in the magnified insert. White dashed lines facilitate comparison of the diaphragm position. Average 4D-Gridded, 4D-Dracula and 4D-MoCo observer scores were 1.0, 2.3 and 3.8 (general image sharpness), 1.0, 2.8 and 3.3 (general streaking artefacts), 1.5, 3.0 and 4.0 (visibility of the tumour extent), 1.5, 2.5 and 3.5 (visibility of the heart) and 0.8, 2.0 and 2.8 (visibility of the oesophagus), respectively.

Dracula-4D networks were each trained in approximately 11 days and took less than 28 seconds to reconstruct high spatio-temporal resolution 4D-MoCo MRI (voxel-size ≈ 1.25 × 1.25 × 3.30 mm^3^, 16 respiratory phases) from 4D-Gridded MRI. For all 12 subjects in the test data, 4D-Dracula image appearance was qualitatively similar to the 4D-MoCo images but exhibited a slight loss of high frequency structures (e.g. small lung vessels). Fig. 3 shows an example comparison between Gridded, Dracula-reconstructed and MoCo images for a lung cancer patient, where heart and tumour extent are similarly visible in Dracula-reconstructed and MoCo images. This is reflected by average observer scores of 3.5 and 3.75 for visibility of the heart and tumour extent, respectively. A similar example for an anaesthetised paediatric patient is provided in the supplemental material. Fig. 4 displays Gridded, Dracula-reconstructed and MoCo images for the case with the lowest observer scores. Minor to moderate blurring is observed in the Dracula-reconstructed images, for instance in the lung, when compared to the corresponding 4D-MoCo images. This finding is supported by the average observer scores for general image sharpness, which were 2.3 and 3.8 for the 4D-Dracula and 4D-MoCo images, respectively. Two movies further illustrating the differences between 4D-Gridded, 4D-Dracula and 4D-MoCo MRI are provided as supplemental material.

The average SSIM value (median, [25th and 75th percentile]) between 4D-Dracula and 4D-MoCo MRI was 0.97 [0.97, 0.98], which was substantially larger compared to the corresponding 4D-Gridded and 4D-MoCo SSIM value: 0.74 [0.59, 0.80]. The average NIQE scores (median, [25th and 75th percentile]) for the 4D-Gridded, 4D-Dracula and 4D-MoCo MRI were 81.03 [56.16, 97.83], 7.94 [6.49, 9.87] and 5.66 [5.16, 9.66], respectively.

Table 1 summarises the scores assigned to inhalation and exhalation images by both observers. For all metrics, 4D-Dracula images were awarded significantly higher scores by both observers when compared to those assigned to the 4D-Gridded images; except by Observer 2 for the delineation of tumour extent metric (*p* = 0.06). The 4D-Gridded images were clinically unacceptable as average scores for the majority of categories were below 2. In contrast, 4D-Dracula images were reported by both observers to be clinically acceptable (mean scores greater than 2) for all metrics and were awarded a total average of 2.7. Although clinically acceptable, 4D-Dracula images were assigned significantly lower scores than the state-of-the-art 4D-MoCo images for most metrics. The reduction in image quality can be observed for a lung cancer patient in Fig. 3, where the 4D-Dracula images, for instance, exhibit minor blurring when compared to the 4D-MoCo images. Scores recorded for the delineation of tumour extent (Observers 1 and 2) and oesophagus visibility metrics (Observer 1) were not significantly different when comparing the 4D-Dracula and 4D-MoCo images. Table 1 additionally displays the ICC values calculated by assessing the scores reported by Observers 1 and 2. For all scores, good inter-observer agreement was determined, except for the general streaking artefact, where excellent agreement was found.

**Table 1:** Scores (mean ± standard deviation) by two experienced observers for 4D-Gridded, 4D-Dracula and 4D-MoCo reconstructed images in a blinded study of eight patients and four healthy volunteers (0 is worst, 4 is best). ^a^ and ^b^ denote statistically significant differences compared to 4D-Gridded and 4D-Dracula, respectively (p < 0.05). The intraclass correlation coefficient (ICC) demonstrates the inter-observer agreement for each score. The Sørensen-Dice coefficient (DSC) was calculated for GTV contours delineated on the 4D-Dracula and 4D-MoCo images. Tumour motion ranges and DSC values are reported as: median [25th percentile, 75th percentile].

Reconstruction algorithm	Gridded		Dracula		MoCo		ICC
Observer number	1	2	1	2	1	2	
General image sharpness (0–4)	1.71 ± 1.04	0.92 ± 0.58	2.96 ± 0.75^a^	2.29 ± 0.91^a^	3.88 ± 0.34^ab^	3.13 ± 0.90^ab^	0.743
Streaking artefacts (0–4)	0.96 ± 0.20	1.00 ± 0.72	2.83 ± 0.56^a^	3.04 ± 0.95^a^	3.50 ± 0.51^ab^	3.71 ± 0.55^ab^	0.840
Delineation of tumour (0–4)	1.60 ± 0.50	1.11 ± 0.33	3.40 ± 0.69^a^	2.22 ± 1.30	3.90 ± 0.32^a^	3.56 ± 0.53^a^	0.747
Heart visibility (0–4)	2.45 ± 0.83	1.30 ± 0.57	3.25 ± 0.64^a^	2.70 ± 1.03^a^	3.95 ± 0.22^ab^	3.50 ± 0.69^ab^	0.612
Oesophagus visibility (0–4)	1.25 ± 0.64	0.45 ± 0.83	2.55 ± 0.76^a^	1.75 ± 1.02^a^	3.00 ± 0.56^a^	3.10 ± 0.72^ab^	0.701
Tumour motion range (mm)	NA	NA	1.6 [1.3, 3.3]	5.8 [1.9, 12.0]	2.2 [2.0, 2.7]	3.5 [1.8, 3.8]	NA
DSC (0–1)	NA	NA	NA	NA	0.89 [0.85, 0.91]	0.87 [0.72, 0.90]	NA

The tumour motion ranges, calculated from the GTV delineations, are included in Table 1. Differences in median tumour ranges, obtained from the 4D-Dracula and 4D-MoCo delineations, were statistically insignificant and were less than 2.4 mm. The median DSC scores, which are also reported in Table 1, between GTV contours delineated on 4D-Dracula and 4D-MoCo images were greater than 0.86 for both observers. The average inter-observer DSC scores for the 4D-Dracula and 4D-MoCo images were (median, [25th and 75th percentile]) 0.83 [0.75, 0.88] and 0.86 [0.81, 0.88], respectively.

**Fig. 5:**
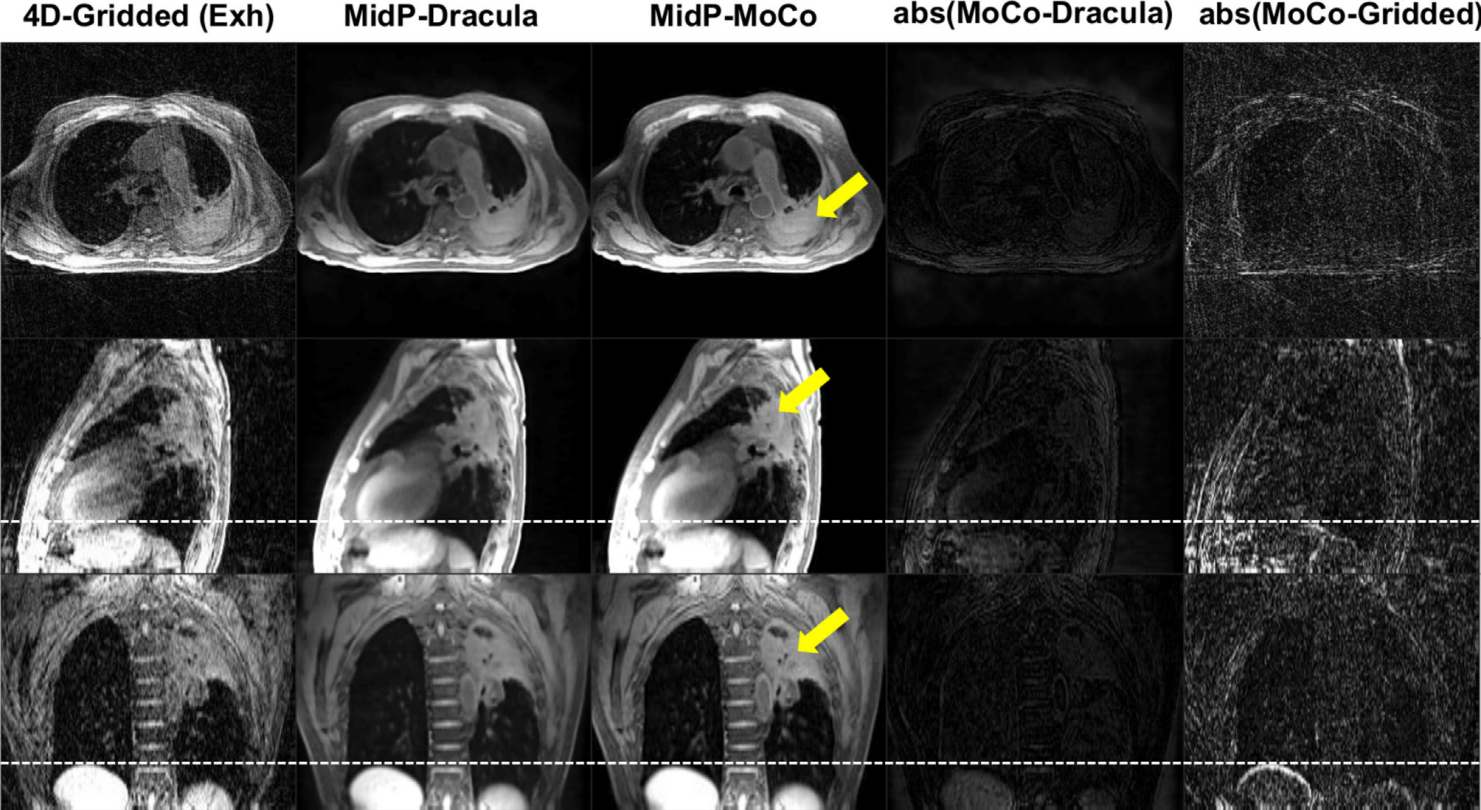
An example comparison of 4D-Gridded (exhalation phase), Dracula-reconstructed midposition (MidP-Dracula) and joint MoCo-HDTV-reconstructed midposition (MidP-MoCo) images for one lung cancer patient. Apart from minor blurring, the appearance of the MidP-Dracula and MidP-MoCo images are similar. White dashed lines aid comparison of the diaphragm surface positions. Yellow arrows point to the tumour site. Window levels in both absolute difference images are the same. Exh: exhalation; MidP: midposition.

Dracula-MidP networks were applied to reconstruct high-resolution MidP-MoCo images from the 4D-Gridded MRI test data of Groups 1 and 2. Training and reconstruction took approximately 11 days and 28 s, respectively. As demonstrated in Fig. 5 for a lung cancer patient, MidP-Dracula images exhibited good visual agreement with the corresponding MidP-MoCo images. In this example, streaking artefacts were greatly reduced in the MidP-Dracula image when compared to the exhalation image of 4D-Gridded MRI. Similarly to the outputs of the Dracula-4D networks, MidP-Dracula images suffered from minor blurring and loss of high-detail information, but maintained a high SSIM value when compared against the MidP-MoCo images: 0.96 [0.94, 0.97]. Moreover, similar NIQE scores were calculated for the MidP-Dracula (8.47 [7.32, 10.13]) and MidP-MoCo images (6.72 [5.04, 9.17]). Excellent agreement was found between tumour positions in the MidP-Dracula and MidP-MoCo images, with a maximum Euclidean distance of 0.63 mm.

## Discussion

Our results demonstrate that it is possible to reconstruct good-quality 4D-MRI with very short reconstruction times. Deep convolutional neural networks were trained and applied for fast reconstruction of 4D-MRI and MidP-MRI from Gridded data (undersampled 4D-MRI). The presented Dracula network required less than 28 s to reconstruct 4D-MRI and MidP images, which is substantially faster than state-of-the-art compressed sensing algorithms, such as GRASP [20], [24] (≈ 10 min) and 4D joint MoCo-HDTV [23] (≈ 9–12 h). 4D-Dracula images were scored significantly better than corresponding 4D-Gridded images and were awarded a mean score (2.7) between adequate (2) and good (3). Using Dracula to calculate the MidP image directly took less time compared to sequential 4D-MRI reconstruction and MidP reconstruction.

A U-net architecture [44] was chosen based on the study by Han et al. [32], which demonstrated the feasibility of applying a 2D U-net to reduce radial streaking artefacts exhibited by undersampled 2D-MRI. This decision was further influenced by the wide range of applications and the availability of a mature open-source implementation. Using a 3D U-net [43] was inspired by modern compressed sensing-based 4D-MRI algorithms [20], [23], [51] which exploit information in both spatial and temporal domains to reconstruct heavily undersampled data. With Dracula’s 3D U-net architecture, spatial information from neighbouring slices was not available, which might have caused blurring of structures experiencing through-plane motion. However, in the presented work it was not possible to implement a 3.5D or 4D U-net architecture due to GPU memory constraints.

Two experienced observers visually assessed the exhalation and inhalation respiratory phase images. They agreed that the 4D-MoCo images had the highest overall image quality with average scores between 3.0 and 4.0 for all metrics, while 4D-Dracula images received scores between 1.8 and 3.4. Both observers reported that minor blurring and residual streaking artefacts exhibited by 4D-Dracula images caused general loss of image quality (sharpness and streaking), but also led to reduced visibility of tumour and organs-at-risk (heart and oesophagus). Nevertheless, 4D-Dracula images were considered clinically acceptable for contouring in radiotherapy treatment planning with the majority of average scores being greater than 2. In contrast, Gridded images were found clinically unacceptable for contouring purposes with average scores between 0.5 and 2.5 for all metrics. Based on the ICC values, good to excellent inter-observer agreement was found between the reported scores.

No statistically significant differences were found between the average tumour motion ranges in 4D-Dracula and 4D-MoCo images. Average tumour motion ranges were based on the delineated contours, which were consistent between 4D-Dracula and 4D-MoCo with average DSC scores above 0.87. Table 1 reveals that the difference in motion range between 4D-Dracula and 4D-MoCo was smaller than the difference in motion range reported by the two observers. While average inter-observer DSC scores were larger than 0.82, which is better than the value of 0.75 reported in [52] for the category of less difficult cases, the interquartile range of the tumour motion extent suggests that the distribution might be skewed. In the case presented in Fig. 4, it was challenging to distinguish between tumour and atelectasis, which led to an average inter-observer DSC score of only 0.38 for this patient.

Similarly to 4D-Dracula MRI, MidP-Dracula images exhibited only minor blurring and residual streaking artefacts when compared to corresponding MidP-MoCo images and had excellent agreement in terms of the SSIM (average value: 0.96). While calculation of SSIM is straight-forward, it is not as sensitive to low contrast features as a trained observer and differs in the assessment of blurred and noisy images [53]. As SSIM requires a reference, we further included the reference-less NIQE metric, which yielded similar average NIQE scores of 8.47 and 6.72 for the MidP-Dracula and MidP-MoCo images, respectively. Finally, the calculated tumour positions in MidP-Dracula exhibited high spatial agreement with those in the MidP-MoCo images, with calculated maximum differences of only 0.63 mm. These results demonstrate the feasibility of rapidly calculating good-quality MidP-MRI directly from 4D-Gridded MRI data.

Dracula could be applied on an MR-Linac, to reconstruct both 4D-MRI and MidP-MRI *online* in less than 1 minute, to support daily plan adaptation for lung and abdominal cancer patients [15], [16], [17]. Alternatively, 4D-Dracula MRI can provide MVFs, which could be used to transform a scan acquired with a separate contrast, to different respiratory phases [54], [55]. This approach yields co-registered 4D-MRI of several contrasts, such as: T1-weighted, T2-weighted and synthetic-CT, without requiring multiple lengthy 4D acquisitions, which could facilitate MR-only radiotherapy treatment planning and delivery on an MR-Linac for lung and abdominal patients [40]. Daily 4D-MRI could inform whether and how motion-management could enable better sparing of normal tissue, for instance by assessing the amplitude of respiratory motion. The supplemental 4D-MRI movies suggest that Dracula is less susceptible to flow effects and high-frequency motion. We suspect that this is a consequence of the variable ratio of cardiac and respiratory frequencies within the training cohort.

Due to the heavy use of image registration in the iterative reconstruction, achieving calculation times of below 10 minutes is challenging for the joint MoCo-HDTV algorithm [23], even with full GPU acceleration. Paulson et al. demonstrated successful use of 4D-MRI in abdominal MR-guided radiotherapy [16], reporting reconstruction times of 3.7 and 6.4 minutes for MidP and 4D-MRI with 8 respiratory frames, respectively. A Dracula-based reconstruction could shorten these reconstruction times and potentially the acquisition time for 4D-MRI, as the joint MoCo-HDTV algorithm [23] can overcome higher undersampling than the CG-SENSE reconstruction used in [16]. Combination of 4D-MRI with imaging during irradiation could yield volumetric real-time MRI either through 2D to 3D deformable registration [13] or signature matching [56], enabling verification of the delivered dose [57], [58].

One limitation of our method is that the 4D-MRI and MidP images used for training are subject to inaccuracies of deformable image registration and to slight over-regularisation of respiratory motion. We expected these to manifest in Dracula-reconstructed images, as Dracula is hypothesised to have learnt any errors present in joint MoCo-HDTV and NiftyReg-calculated data during the training process. Future work will explore model-based deep learning reconstructions, which allow for inclusion of data consistency layers in a deep cascading approach [29]. Model-based approaches potentially could reduce errors by promoting data fidelity. Treating the joint MoCo-HDTV algorithm as a variational network [28] might enable MVFs (applied for reconstruction purposes in [23]) to be directly calculated without requiring a separate deformable image registration step, potentially resulting in reduced errors. A second limitation relates to the mean square error loss metric, which might not be sensitive enough to resolve the large dynamic range of MR images. Recently a feature-wise perceptual loss was found to outperform pixel-wise (L1 and L2) and patch-wise loss functions in cardiac MR imaging [59]. The improvements in image quality found between epochs 20 and 120 are not reflected in the validation loss, which appeared stable. We expect that further training (i.e. epochs >120) would result in overfitting, but this hypothesis was not tested due to the considerable time required to train each network. Another limitation of our work is that we only performed a 2-fold cross validation. While testing on the whole patient cohort would be possible, the considerable resource requirements for training and scoring prevented us from doing so. A further limitation of this study is that only data acquired on a diagnostic MRI scanner was evaluated. This was due to the limited availability of sufficient 4D-MRI data sets to train an MR-Linac specific version of the Dracula reconstruction. Future work will focus on training and applying the Dracula reconstruction to MR-Linac data directly, hypothesising that this will lead to significant improvements in image quality and usability over 4D Gridded data and enable ultra-fast 4D-MRI and MidP image reconstruction for MR-guided radiotherapy.

In conclusion, Dracula neural networks were separately trained and applied to reconstruct 4D-MRI and MidP-MRI from Gridded data in only 28 seconds. Dracula-reconstructed MRI had excellent agreement with corresponding joint MoCo-HDTV-reconstructed MRI (SSIM ≥ 0.96) and were considered clinically acceptable for target and OAR delineation. Rapidly calculated 4D-MRI and MidP-MRI could provide up-to-date information to support radiotherapy treatment plan adaptation in thoracic and abdominal MR-Linac workflows.

## Sources of support

We gratefully acknowledge the support of NVIDIA Corporation with the donation of the Quadro P6000 GPU used for this research.

We acknowledge NHS funding to the NIHR Biomedical Research Centre and the Clinical Research Facility at The Institute of Cancer Research and The Royal Marsden NHS Foundation Trust. We acknowledge funding from CR UK programme grants C33589/A28284 and C7224/A23275, and from project grant C309/A20926.

## Conflict of interest

The Institute of Cancer Research and the Royal Marsden NHS Foundation Trust are part of the Elekta MR-Linac Research Consortium. Joshua Freedman is currently an employee at Elekta. However, the work described in this article was fully completed while Joshua Freedman was a PhD candidate at The Institute of Cancer Research and the Royal Marsden NHS Foundation Trust.
