# Supplementary material for: Rapid 4D-MRI reconstruction using a deep radial convolutional neural network: Dracula
Source: Radiother Oncol. 2021 Jun;159:209–17. doi: 10.1016/j.radonc.2021.03.034 (PMC8216429; doi:10.1016/j.radonc.2021.03.034)
Supplement: Supplementary data 1 [file mmc1.docx]

**Supplemental Material: Acquisition Parameters**

Displays a detailed overview of the acquisition parameters used to acquire the radial stack-of-stars sequence.

| **Parameter** | **Radial stack-of-stars** |
| --- | --- |
| Orientation | Axial |
| Number of slices | 64 - 96 |
| Slice oversampling, % | 20.0 - 27.7 |
| Number of spokes per slice | 1005 - 1008 |
| Acquisition time, min | 04:21 - 05:45 |
| In-plane field-of-view, mm^2^ | 400 × 400 - 480 × 480 |
| Pixel size, mm^2^ | 1.25 × 1.25 -1.50 × 1.50 |
| Slice thickness, mm | 3.0 - 3.5 |
| Echo time, ms | 1.48 - 1.57 |
| Repetition time, ms | 3.18 |
| Flip angle, º | 8 |
| Pixel bandwidth, Hz | 630 |

**Table 1:** Acquisition parameters of the radial stack-of-stars sequence.

**Supplemental Material: Dracula Implementation Details**

Convolutional layers (light blue arrows in Figure 1 in the manuscript) consisted of multiple convolution, non-linear activation and batch normalisation functions. Convolution operators were applied with kernel-size 3 × 3 × 3 and zero-padding. All kernel weights were initialised using a Glorot normal distribution [1]. Each convolution operation resulted in a feature map. For instance, in the first layer, the input image was operated on by 64 independent convolutions, which resulted in 64 feature maps. Following convolution, each generated feature map was operated on by a ReLu activation function, which set all negative values in the feature map to zero. After non-linear activation, batch normalisation was used to normalise the output values of the activated feature maps [2]. Note that scaling and shifting parameters applied in batch normalisation were trainable.

Downsampling layers (red arrows in Figure 1) were implemented using a max pooling operator with kernel-size 2 × 2 × 2. Downsampling reduced the matrix-size of input feature maps by a factor of 2 in each dimension. Downsampled feature maps are represented by red blocks in Figure 1.

Upsampling layers (black arrows in Figure 1) were applied using a transposed convolution operator with kernel-size 2 × 2 × 2 [3]. Similarly to the convolutional layers, Glorot normalisation was employed to initialise kernel weights in the transposed convolutional operators. Upsampling doubled the matrix-size of input feature maps in each dimension. Skip connections (dark blue arrows in Figure 1) were incorporated by copying and concatenating feature maps from the encoding path (blue blocks in Figure 1) with feature maps of the same matrix-size in the decoding path (black blocks in Figure 1) [4]. The final convolutional layer (purple arrow in Figure 1) applied one convolution operation then non-linear activation (sigmoid function) to feature maps obtained following the decoding path. The convolution was applied with kernel-size 1 × 1 × 1.

Drop-out was employed after all downsampling and upsampling layers to reduce overfitting [5]. Drop-out refers to randomly setting a fraction of layer outputs to 0 during each training update. The drop-out fraction was set to 1/2, except for the first downsampling layer, which was set to 1/4.

The encoding path consisted of eight convolutional and four downsampling layers. Feature maps with increasingly reduced matrix-size were calculated by passing the input image through layers in the encoding path. Low matrix-size feature maps calculated along the encoding path contained high-level representations of the input data. The bottle-neck path connected feature maps from the encoding path to the decoding path; it contained two convolutional layers. The decoding path included eight convolutional and four upsampling layers. Skip connections permitted information from the encoding path to be accessed by the decoding path. A final convolutional layer was implemented to extract a single image from all feature maps output by the decoding path. The matrix-size of the output image had the same dimensions as the input image.

References:

[1] Glorot X, Bengio Y. Understanding the difficulty of training deep feedforward neural networks. AIStats2010. p. 249-56.

[2] Ioffe S, Szegedy C. Batch normalization: Accelerating deep network training by reducing internal covariate shift. arXiv preprint arXiv:150203167. 2015.

[3] Çiçek Ö, Abdulkadir A, Lienkamp SS, Brox T, Ronneberger O. 3D U-Net: learning dense volumetric segmentation from sparse annotation. MICCAI 19th meeting: Springer; 2016. p. 424-32.

[4] Ronneberger O, Fischer P, Brox T. U-net: Convolutional networks for biomedical image segmentation. International Conference on Medical image computing and computer-assisted intervention: Springer; 2015. p. 234-41.

[5] Srivastava N, Hinton G, Krizhevsky A, Sutskever I, Salakhutdinov R. Dropout: a simple way to prevent neural networks from overfitting. J Mach Learn Res. 2014;15:1929-58.

**Supplemental Material: Paediatric Example**

The figure below displays an example comparison between Gridded, Dracula-reconstructed and MoCo images for a representative paediatric patient under anaesthesia.


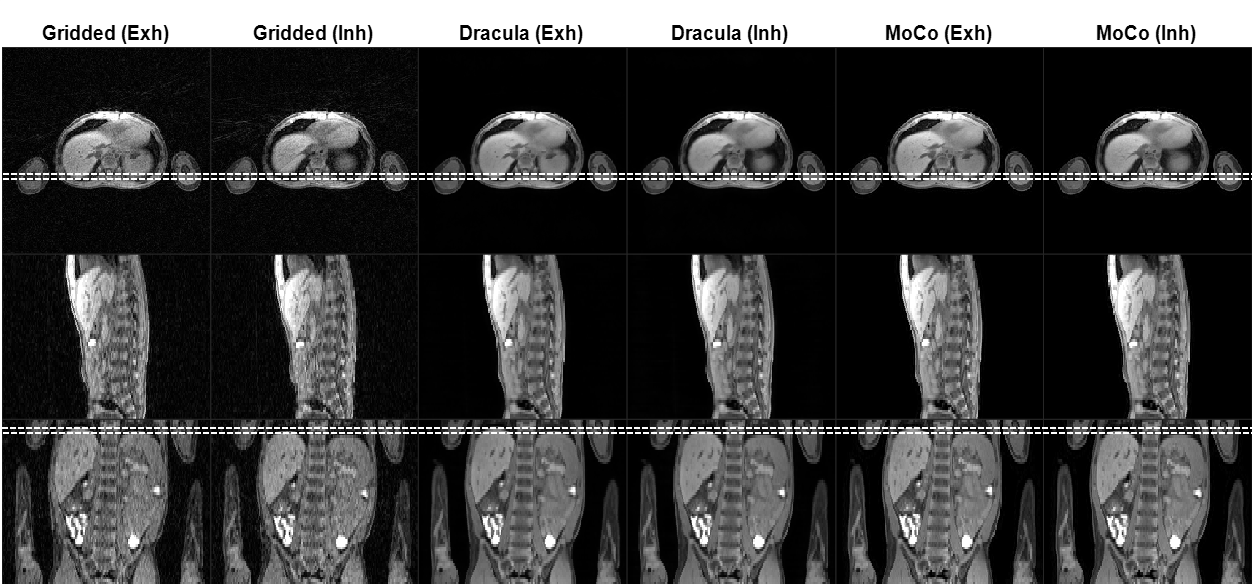


Fig. 1: Comparison of the exhalation (Exh) and inhalation (Inh) respiratory phases of the 4D-Gridded, 4D-Dracula and 4D-MoCo reconstructed images of a representative anesthatised peadiatric patient. Dracula restores image quality in a comparable manner to the joint MoCo-HDTV reconstruction with only minor blurring. White dashed lines aid comparison of the diaphragm position.
