# Supplementary figures and images for: Rapid 4D-MRI reconstruction using a deep radial convolutional neural network: Dracula
Source: Radiother Oncol. 2021 Jun;159:209–17. doi: 10.1016/j.radonc.2021.03.034 (PMC8216429; doi:10.1016/j.radonc.2021.03.034)

## Slide 1
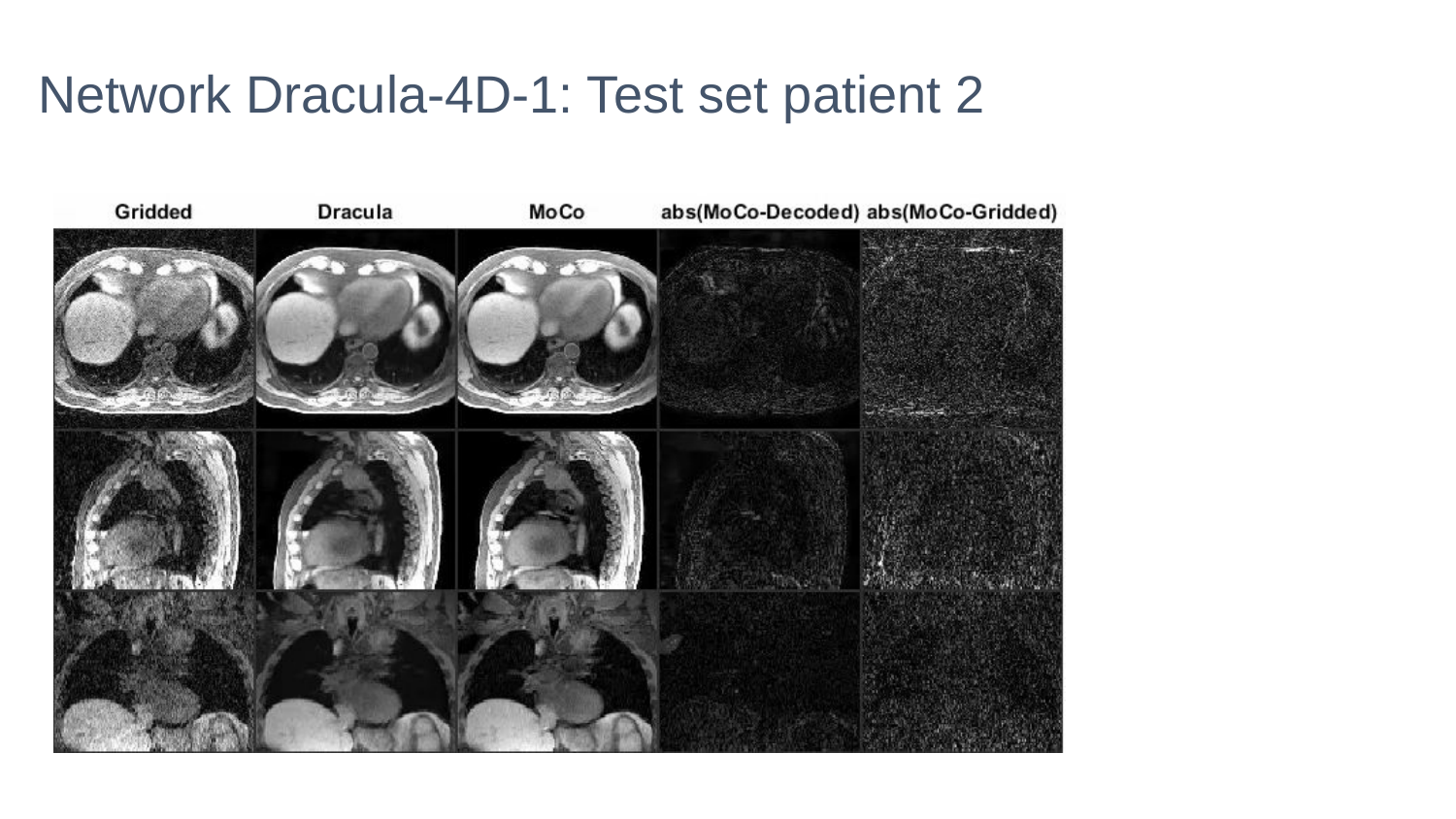

Network Dracula-4D-1: Test set patient 2

## Slide 2
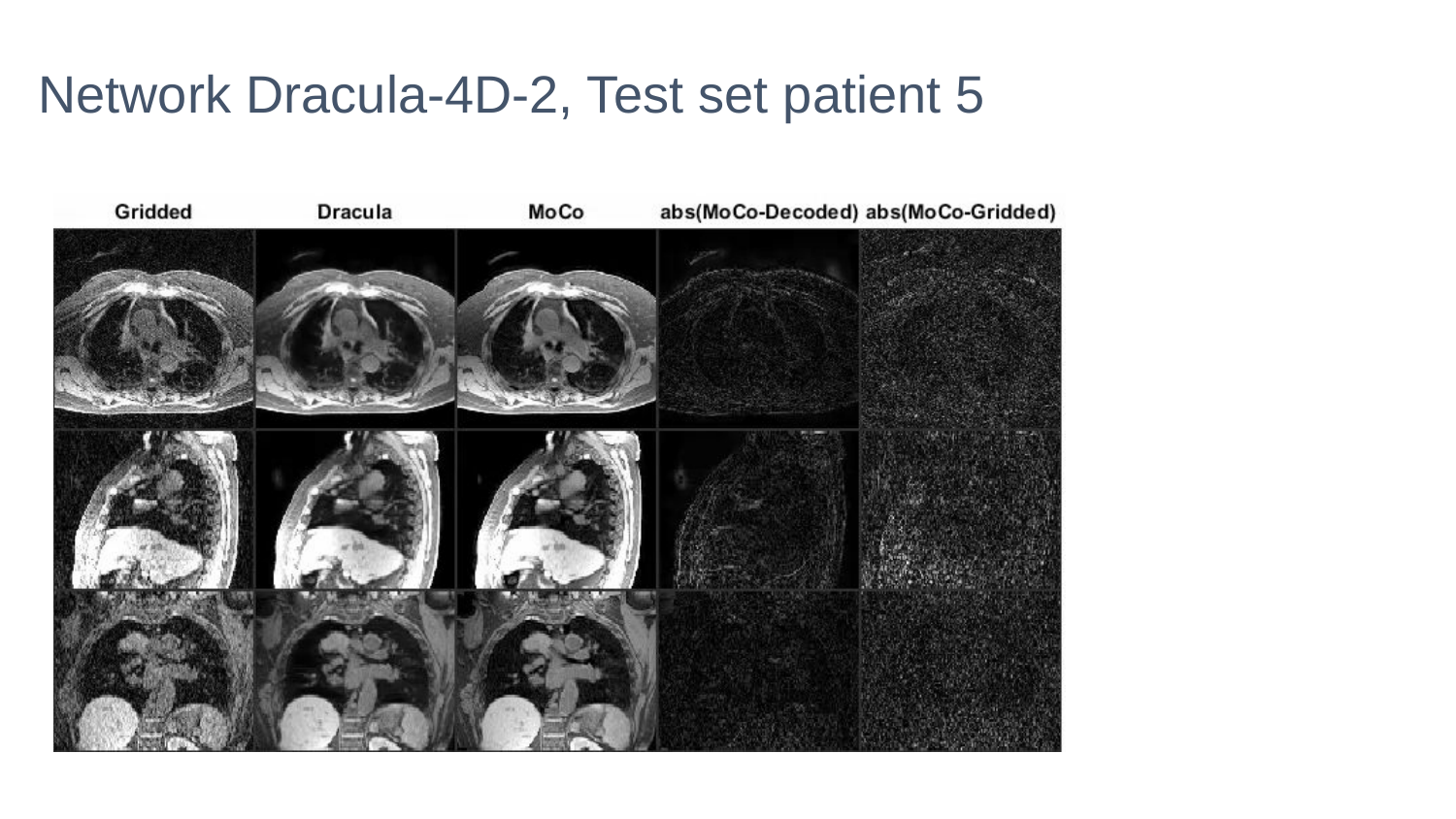

Network Dracula-4D-2, Test set patient 5

Supplement: Supplementary data 2 [file mmc2.pptx]
